# Early-life gammaherpesvirus infection results in an expanded long-term latent reservoir

**DOI:** 10.3389/fcimb.2026.1874370

**Published:** 2026-07-15

**Authors:** Mikayla S. Manzi, Lufuno Phophi, Julianne Montes F. de Oca, Haley M. Wilt, Karl J. Ensberg, April Feswick, Stephanie M. Karst, Scott A. Tibbetts

**Affiliations:** Department of Molecular Genetics and Microbiology, UF Health Cancer Institute, UF Genetics Institute, College of Medicine, University of Florida, Gainesville, FL, United States

**Keywords:** early life infection, Epstein-Barr virus (EBV), herpesvirus, latency, murine γ-herpesvirus 68 (MHV68), virus

## Abstract

Gammaherpesviruses are ubiquitous oncogenic pathogens that establish lifelong latency, are associated with multiple types of malignancies, and are increasingly believed to play a role in autoimmune disease. Human Epstein–Barr virus (EBV) and Kaposi sarcoma-associated herpesvirus (KSHV) frequently infect across various stages of childhood development. Nevertheless, how infection during the earliest stages of life, when immune defenses are still developing, shapes the risk of malignant disease and autoimmunity remains poorly understood. Here, we characterized acute and chronic infection following early-life inoculation with murine gammaherpesvirus 68 (MHV68). We infected mice at postnatal day P3, P5, or P8, ages that, in the compressed developmental timeline of the mouse model, correspond to the progressive immune maturation that occurs in human neonatal to infancy stages. Mice infected during early life displayed age- and dose-dependent susceptibility to MHV68 infection, with the approximate lethal dose 50% (LD50) values ranging from 200 plaque forming units (PFU) in mice inoculated at P3 to 10,000 PFU in mice inoculated at P8. Severe disease was accompanied by substantial weight loss prior to death. Following intranasal inoculation of P8 pups, MHV68 replicated rapidly in the lung, reaching high titers by 3 days, achieving higher lung titers than adult mice at 5 days. Nevertheless, acute replication was largely resolved by 16 days in surviving animals. Early-life infection induced splenomegaly and resulted in the establishment of latency at frequencies similar to those of adult mice. In contrast, mice infected as neonates maintained 14-fold higher frequencies of latently infected cells than adults during the long-term stable phase of latency, months after clearance of a lytic infection, suggesting that early-life infection substantially alters the long-term latent reservoir. Together, these data demonstrate for the first time that gammaherpesvirus exposure during early life imprints a long-lasting increase in latency burden and establishes a foundation to determine potential implications for malignancy and autoimmune risk later in life.

## Introduction

The human gammaherpesviruses, Epstein–Barr virus (EBV) and Kaposi sarcoma-associated herpesvirus (KSHV), are ubiquitous oncogenic pathogens that account for a large number of malignancies worldwide, including in children as young as 2 years (Molyneux et al., 2012; Jackson et al., 2016; El-Mallawany et al., 2018). EBV infects up to 95% of the human population (Young et al., 2016), whereas KSHV prevalence varies geographically, with particularly high rates of infection in sub-Saharan Africa (Motlhale et al., 2022; Mamimandjiami et al., 2025). Although gammaherpesvirus infections are typically asymptomatic during early stages, the establishment of lifelong latency provides an opportunity for these viruses to promote malignancy or autoimmune disease years after initial infection. For example, EBV is linked to multiple malignancies and immune-mediated diseases, including Hodgkin lymphoma, diffuse large B-cell lymphoma, nasopharyngeal carcinoma, and gastric carcinoma, and has recently been linked to multiple sclerosis (Shannon-Lowe and Rickinson, 2019; Bjornevik et al., 2023; Leung et al., 2023). KSHV is the causative agent of Kaposi sarcoma (KS), multicentric Castleman disease, primary effusion lymphoma, and Kaposi sarcoma inflammatory cytokine syndrome (KICS) (Bhutani et al., 2015).

Although gammaherpesvirus infections are commonly associated with the development of disease decades after initial exposure to the virus, in some settings infection is associated with pediatric malignancy. These include Burkitt lymphoma (BL) and childhood KS, both of which are aggressive cancers that commonly present in children 6 to 11 years of age (Molyneux et al., 2012; Jackson et al., 2016). In affected regions, childhood BL and KS represent substantial fractions of pediatric cancer cases, often among the most commonly observed childhood malignancies (Stefan, 2015). BL is classified into endemic, sporadic, and immunodeficiency-associated subtypes (Molyneux et al., 2012). Endemic BL, which has a high incidence in sub-Saharan Africa, Papua New Guinea, and some regions of South America, is consistently EBV-positive and is strongly associated with malaria *Plasmodium falciparum* co-infection, with the highest incidence of BL occurring in malaria holoendemic regions (Brady et al., 2008; Molyneux et al., 2012; Hämmerl et al., 2019). Pediatric KS, which predominantly occurs in sub-Saharan Africa, is commonly distinguished by HIV status, resulting in endemic (HIV-negative) and epidemic (HIV-positive) subtypes (Jackson et al., 2016). KS can also present differently in children as compared to adults, with aggressive disease with lymph node and mucosal site involvement seen more frequently in children (Caro-Vegas et al., 2023). Thus, pediatric gammaherpesvirus-associated diseases differ from adult disease manifestations. Notably, though, the mechanisms underlying these differences remain poorly understood.

In addition to the important cofactors of malaria and HIV, epidemiologic studies have correlated childhood malignancy risk with gammaherpesvirus infection during early childhood, particularly before 2 years of age (Guy de The, 1977; Moormann et al., 2005; Chabay and Preciado, 2013). Globally, most individuals acquire EBV or KSHV during childhood or adolescence, and regions with high rates of pediatric gammaherpesvirus-associated malignancies also exhibit high rates of early childhood infection (Piriou et al., 2012). That said, most prior studies have focused extensively on defining age at primary infection or identifying cofactors associated with malignancy (Kasolo et al., 1997; Moormann et al., 2009; Minhas et al., 2010; Piriou et al., 2012; Slyker et al., 2013; Jayasooriya et al., 2015; Sabourin et al., 2021; Sabourin et al., 2023). Substantially less is known about how infection during infancy or early childhood alters gammaherpesvirus pathogenesis, in large part due to the difficulty of enrolling children in long-term studies, the biological complexity introduced by cofactors, and the lack of tractable experimental infection models.

Murine gammaherpesvirus 68 (MHV68, MuHV-4, γHV68), a natural pathogen of rodents originally isolated from bank voles, has been extensively used as a model for chronic gammaherpesvirus infection (Simas and Efstathiou, 1998; Virgin and Speck, 1999; Speck and Virgin, 1999; Barton et al., 2011; Wang et al., 2021). MHV68 is genetically and pathogenically related to EBV and KSHV, establishes lifelong latency in B cells, and is associated with B-cell malignancies (Sunil-Chandra et al., 1994; Tarakanova et al., 2005), thus providing a highly tractable system for dissecting *in vivo* gammaherpesvirus infection and pathogenic determinants. While this system provides an excellent opportunity for examining the consequences of early-life infection on chronic infection and pathogenesis, the vast majority of MHV68 studies have been performed in adult mice (7 weeks or older).

Human developmental terminology generally defines the neonatal period as birth through 28 days of life, with infancy as 1 to 12 months, early childhood as 1 to 5 years, middle childhood as 6 to 10 years, and adolescence as 11 to 18 years. However, in murine systems, the term “neonatal” typically refers to the full postnatal period prior to weaning from the mother, typically 21 days after birth. In mice, postnatal development is greatly compressed; thus, murine neonatal ages such as postnatal days P3, P5, and P8 do not correspond to specific human developmental phases, but instead represent progressive human neonatal to early childhood stages during which innate and adaptive immune responses are still maturing (Basha et al., 2014; IJspeert et al., 2016; Yu et al., 2018; Nielsen et al., 2019; Semmes et al., 2021). For the purpose of this manuscript, we will refer to infection of P3, P5, or P8 mice as infections of neonatal mice, with an eye toward early-life human infections that occur from birth through infancy.

Neonatal mouse infection models reflecting these progressive immunological development stages have been used successfully to study other acute and chronic virus infections relevant to childhood disease (Bonville et al., 2010; You et al., 2015; Yin et al., 2021; Peiper et al., 2023; Mccord et al., 2026). Although previous studies have examined some aspects of neonatal MHV68 infection (Häusler et al., 2007; Ptaschinski and Rochford, 2008; Štiglincová et al., 2011), early-life gammaherpesvirus infection remains incompletely characterized, particularly with regard to the impact of early-life exposure to the virus on the lifelong latency reservoir and long-term disease. In the work presented here, we characterized lytic replication, acute disease, latency establishment, and long-term latency maintenance following early-life infection, finding that animals that are exposed to the virus during immunological development stages exhibit a substantially elevated latency load during stable long-term infection.

## Materials and methods

### Mice and infections

C57BL/6J dams and pups were housed at the University of Florida (Gainesville, FL) in accordance with all federal and university guidelines. All animal protocols were approved by the Institutional Animal Care and Use Committee at the University of Florida. Pups were inoculated intraperitoneally (i.p.) with 50 µL or intranasally (i.n.) with 8 µL under isoflurane anesthesia with a micropipette. Adults were infected intranasally with 20 μL. No death was observed following anesthesia of the pups. For i.n. inoculations, animals were anesthetized via vaporizer, providing 3%–5% isoflurane until loss of the righting reflex, then quickly infected, placed back into their cage, and observed until normal behavior was detected. Pups were inoculated on postnatal day P3, P5, or P8 with mock inoculum or with wild-type MHV68, MHV68.H2bYFP (Collins et al., 2009; Collins and Speck, 2012), or MHV68.ORF73βla (Nealy et al., 2010) (phenotypically wild-type recombinant marker viruses), which were diluted in serum-free Dulbecco's Modified Eagle Medium (DMEM) over a dose range of 50 to 10,000 PFU. Pups were weighed daily until P25 or euthanized earlier if they reached humane endpoint body scoring criteria. Animals were euthanized using carbon dioxide gas at a displacement rate of 30%–70% of the chamber volume per minute. For long-term experiments (25 or more days), pups were weaned on P25 and separated by sex. At least three independent litters were infected at each age and dose-tested. Litter size was four to eight pups for all experiments.

### Plaque assay

Lungs were harvested 3 to 16 days post-infection (dpi) and frozen at −80 °C in 2-mL screw-top tubes (Heathrow Scientific, 10060, Vernon Hills, IL) containing 1 mL serum-free DMEM and 1.0-mm zirconia/silica beads (BioSpec Products, 11079105Z, Bartlesville, OK). On the day of plaque assay, tubes were thawed on ice and subjected to mechanical disruption using a Mini-Beadbeater (BioSpec Products, Bartlesville, OK). Lungs were disrupted for 2 min at the highest speed, placed on ice for 2 min, and then disrupted again for 2 min. Samples were centrifuged for 1 min at 2,000 rpm, and supernatant was transferred to 2-mL cryogenic tubes for long-term storage (Corning, 430659, Corning, NY) and used in plaque assays as previously described (Van Dyk et al., 2000; Tibbetts et al., 2003b; Wang et al., 2023).

### Limiting dilution nested PCR assay

Following the inoculation of P5 or P8 pups or 8–12-week-old adults, spleens were harvested at 16, 42, and 90 days. Spleens were mechanically disrupted, red blood cells were lysed with red blood cell lysis buffer (0.144 M ammonium chloride, 0.017 M Tris, pH 7.2), and remaining cells were strained through a 100-µm nylon cell strainer (Corning, 431752, Corning, NY). Splenocytes were frozen in 80% heat-inactivated fetal bovine serum and 20% Dimethyl Sulfoxide (DMSO) and kept at −80 °C. Samples were thawed, and resuspended splenocytes were serially diluted in a background of RAW 264.7 macrophages such that each final dilution resulted in reactions containing 10,000 total cells each. Cells were added to 96-well plates (Eppendorf E951020362, Hamburg, Germany) at 12 wells per cell dilution, as previously described (Weck et al., 1999; Van Dyk et al., 2000; Tibbetts et al., 2003b; Coleman et al., 2010; Wang et al., 2023). Cells were lysed with proteinase K for 8 h at 56 °C, followed by inactivation for 20 min at 95 °C. Two rounds of PCR targeting the MHV68 gene ORF72 were performed, and the 195-bp product was visualized on a 3% agarose gel. The primers for round 1 PCR were 5′-GAGATCTGTACTCAGGCACCTGT-3′ and 5′-GGATTTCTTGACAGCTCCCTG-3′. The primers for round 2 PCR were 5′-TGTCAGCTGTTGTTGCTCCT-3′ and 5′-CTCCGTCAGGATAACAACGTC-3′.

### *Ex vivo* reactivation and preformed virus assays

Single-cell splenocyte suspensions from limiting dilution PCR analyses were serially diluted in parallel and plated onto murine embryonic fibroblasts that had been plated 24 h earlier, as previously described (Weck et al., 1999; Van Dyk et al., 2000; Tibbetts et al., 2003b; Coleman et al., 2010; Wang et al., 2023). Plates were incubated for 21 days and then analyzed for cytopathic effect (CPE) resulting from reactivating MHV68. To control for preformed infectious virus, parallel cell suspensions were resuspended in one-third DMEM with 0.5-mm zirconia/silica beads and disrupted using a Mini-Beadbeater (BioSpec Products, Bartlesville, OK). This process lyses cells while leaving the preformed infectious virus intact. Samples were centrifuged, separated from beads, serially diluted, and plated onto murine embryonic fibroblasts. Plates were incubated for 21 days and assessed for CPE.

### Statistical analyses

All data were analyzed using the Prism 11 software (GraphPad, San Diego, CA). Statistical significance was determined using two-tailed, unpaired Student’s t-tests for comparisons between pups and adult titers, Brown–Forsythe and Welch’s ANOVA with Dunnett’s T3 multiple comparisons for spleen weight comparison between multiple groups, and the Wilcoxon signed-rank test when viral titers were below the limit of detection. Data were visualized using a Q-Q plot for normality prior to parametric analysis (due to the very large sample sizes required for standard normality tests). The log-rank (Mantel–Cox) test was used to determine significance from Kaplan–Meier survival curves. As mice succumbed to infection at varying time points throughout experiments, significance between weight experimental groups was determined using a mixed-effects model, as a repeated-measures ANOVA is unable to handle missing values. This mixed model uses a compound symmetry covariance matrix and is fit using restricted maximum likelihood (REML). When values are missing (missing completely at random), the results can be interpreted like repeated-measures ANOVA. Column effects were compared to determine significance in weight gain between groups. p-Values less than 0.05 were considered statistically significant. Significance is indicated as follows: ns, not significant; *p < 0.05; **p < 0.01; ***p < 0.001.

## Results

### Age at inoculation determines susceptibility to MHV68 infection in neonatal mice

**Figure 1:**
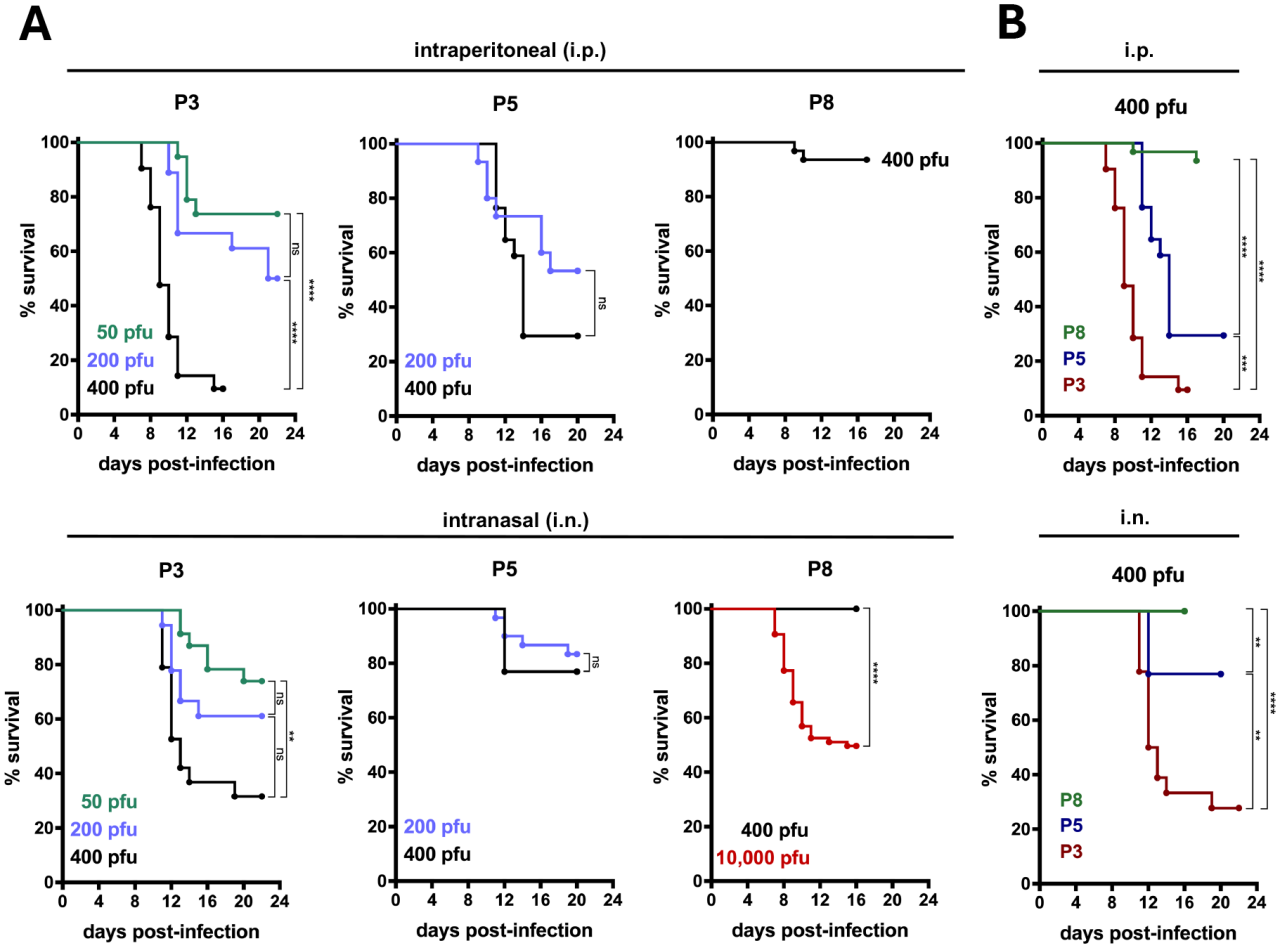
Age at inoculation determines susceptibility to MHV68 disease following early-life infection. **(A)** Pups were inoculated i.p. (top) or i.n. (bottom) on P3, P5, or P8 with the indicated doses of MHV68, and survival was monitored until P25. Each line represents infection of at least three independent litters, with four to eight pups per litter. ***p < 0.001; ****p < 0.0001; ns, not significant. **(B)** Survival in pups following inoculation with 400 PFU MHV68, stratified by postnatal age at inoculation. Each line represents infection of at least three independent litters with four to eight pups per litter. **p < 0.01; ****p < 0.0001; ns, not significant. MHV68, murine gammaherpesvirus 68; i.p., intraperitoneal; i.n., intranasal.

Because early-life MHV68 infection has not been extensively characterized, we first tested key parameters such as age at inoculation, virus dose, and the route of inoculation in order to identify conditions that would permit careful longitudinal analysis of chronic infection. Mouse pups were infected on postnatal day 3, 5, or 8 (P3, P5, or P8, respectively) to include a range of ages within the neonatal period. Pups were inoculated either i.n. or i.p., and survival was monitored until P25 (**Figure 1A**). Across the tested conditions, pups displayed age- and dose-dependent susceptibility to MHV68 infection. Animals inoculated at postnatal day 3 were the most susceptible to lethal infection, with a lethal dose 50% (LD50) of approximately 200 PFU, and a subset of animals demonstrated lethality at doses as low as 50 PFU following both i.p. and i.n. inoculations. In contrast, P8 animals were resistant to lower doses of infection, demonstrating an LD50 of approximately 10,000 PFU. The analysis of pups inoculated across groups with 400 PFU allowed for a clear comparison of postnatal age-dependent susceptibility. While P8 pups inoculated with 400 PFU virus were nearly 100% resistant to lethality, nearly 100% of P3 pups inoculated with 400 PFU virus succumbed to infection, with the vast majority of deaths occurring 8–11 days post-infection (dpi) following i.p. inoculation, and 11–13 dpi following i.n. inoculation (**Figure 1B**). Together, these data demonstrate that postnatal age at inoculation is a major determinant of neonatal susceptibility to gammaherpesvirus infection.

### Severe neonatal MHV68 disease is accompanied by weight loss

**Figure 2:**
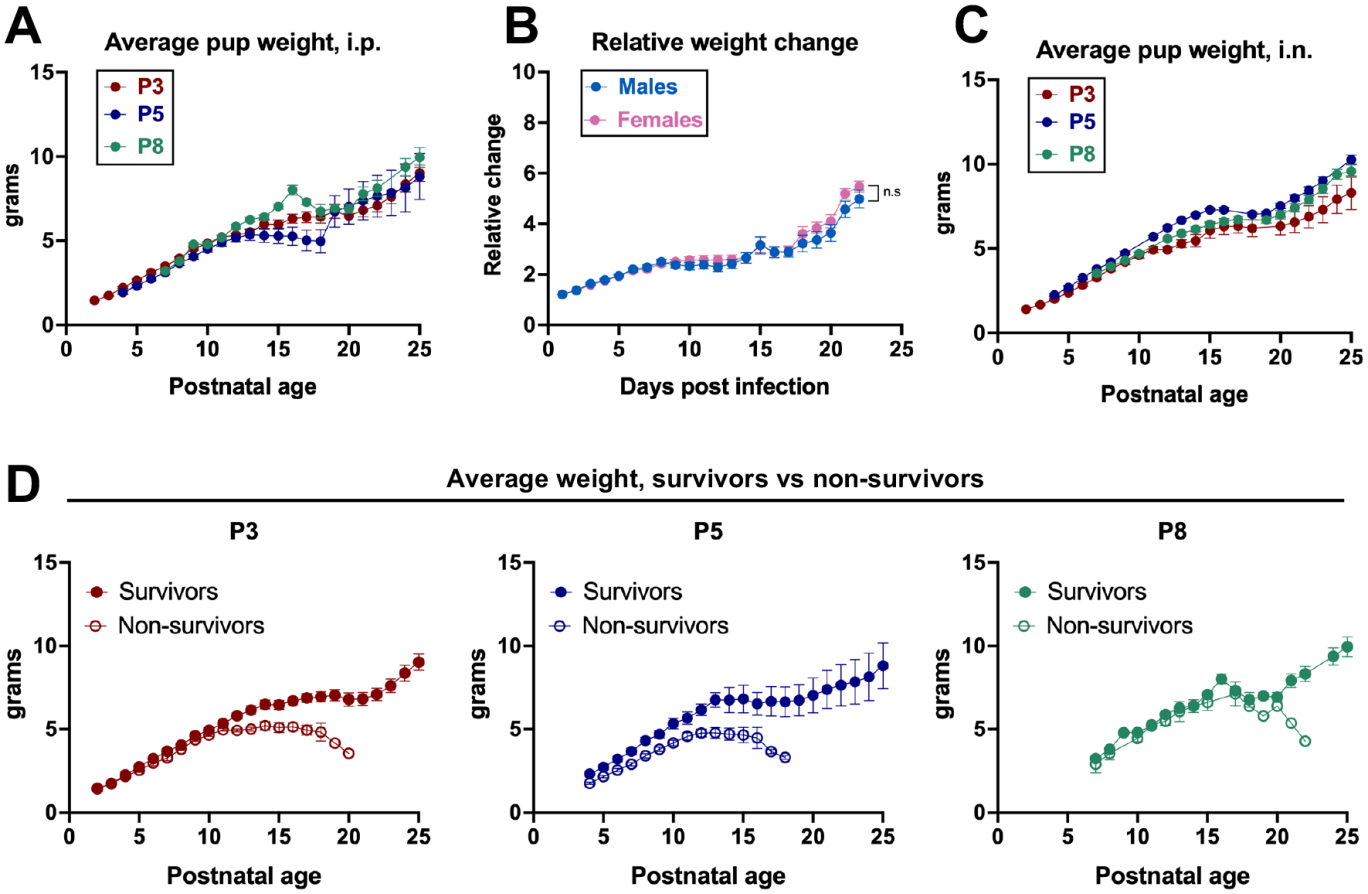
Severe disease during early life infection is accompanied by weight loss. **(A)** Average body weight of pups following i.p. inoculation with 400 PFU MHV68, stratified by postnatal age at time of inoculation. Symbols represent mean ± SEM (P3, n = 23; P5, n = 17; P8, n = 20). **(B)** Relative weight change in male and female pups following i.p. inoculation with 400 PFU MHV68. Relative weight change was calculated from initial starting weight, which was 1 day prior to infection. Significance was determined using mixed-effect analysis comparing column effect (sex) [F (1,68) = 0.1108, p = 0.7402] (male, n = 32; female, n = 38). **(C)** Average body weight of pups following i.n. inoculation with 400 PFU MHV68, stratified by postnatal age at time of inoculation. Symbols represent mean ± SEM (P3, n = 20; P5, n = 20; P8, n = 11). **(D)** Average body weight of pups that survived or did not survive following i.p. inoculation with 400 PFU MHV68, stratified by postnatal age at inoculation. Symbols represent mean ± SEM (P3 survivors, n = 9; non-survivors, n = 14; P5 survivors, n = 5; non-survivors, n = 12; P8 survivors, n = 18; non-survivors, n = 2). MHV68, murine gammaherpesvirus 68; i.p., intraperitoneal; i.n., intranasal.

To identify clinical features associated with neonatal MHV68 disease, we monitored body score and weighed pups daily following inoculation at 400 PFU. Among all postnatal day inoculation groups, we observed no significant differences in average weight gain throughout the experimental window (**Figure 2A**). Additionally, we observed no significant difference across all groups when comparing male versus female animals (**Figure 2B**), or when the virus was administered intranasally (**Figure 2C**). Notably, though, the stratification of non-survivors demonstrated that in pups in which infection was lethal, severe disease and substantial weight loss occurred prior to death, regardless of postnatal age at time of inoculation (**Figure 2D**).

### Neonatal MHV68 infection results in rapid and sustained lytic replication in the lung

**Figure 3:**
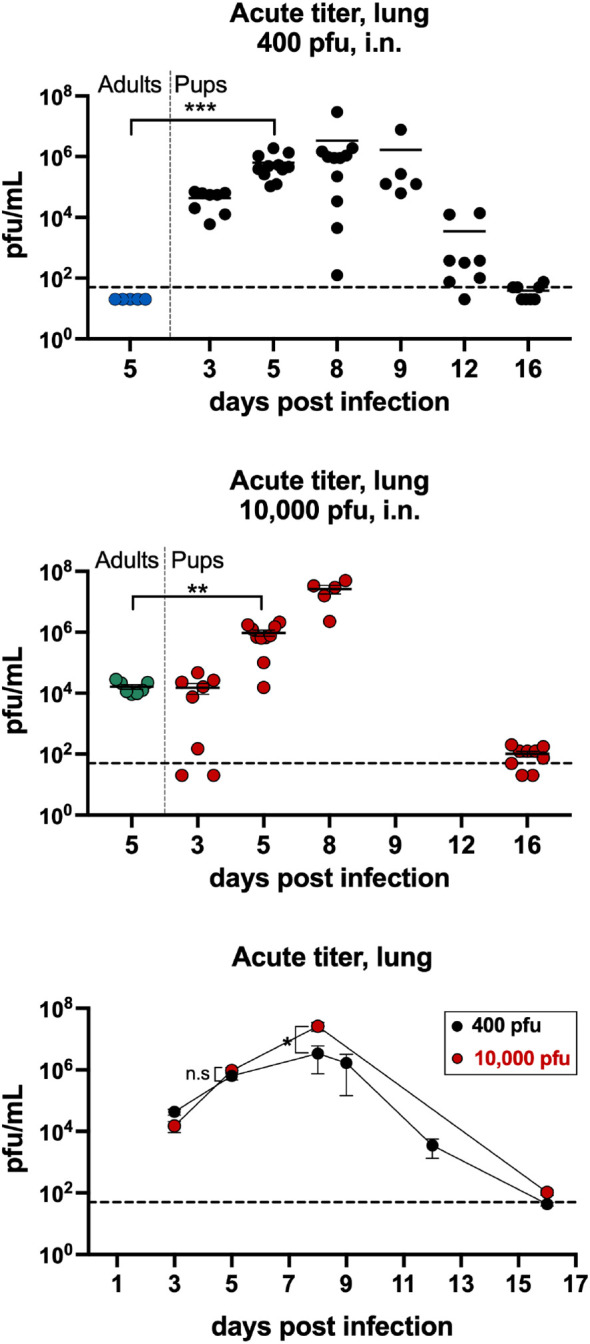
Early-life infection results in rapid and elevated lytic replication in the lung. **(A, B)** P8 pups were inoculated i.n. with **(A)** 400 PFU or **(B)** 10,000 PFU MHV68. Adult mice were inoculated in parallel, where indicated. Lungs were harvested at the indicated time points, and a plaque assay was performed to quantify infectious virus titer. All titers were normalized to homogenate volume. Each symbol represents an individual mouse. Significance was determined using the Wilcoxon signed-rank test **(A)** and a two-tailed, unpaired Student’s t-test **(B, C)** Mean lung virus titers over time following early-life inoculation. The dotted line at y = 50 indicates the limit of detection (LOD). Significance was determined using a two-tailed, unpaired Student’s t-test on time points with all samples from both doses above LOD. *p < 0.05; **p < 0.01; ns, not significant. MHV68, murine gammaherpesvirus 68; i.n., intranasal.

In adult mice, i.n. inoculation results in acute virus replication in the lungs, which typically peaks between 4 and 7 days and resolves by 16 days (Sunil-Chandra et al., 1992). To characterize the acute phase of infection in neonatal mice, we quantified virus titers in the lungs at multiple time points over the 16-day time course. Because P8 pups inoculated i.n. with 400 PFU survived at high frequency, we initially used this infection condition to characterize lytic replication without substantial loss of animals. In the neonatal setting, virus titers increased rapidly, reaching high titers (mean 4 × 10e4 PFU/mL) as early as 3 days and continuing to increase until peaking by 8 dpi (mean 3.4 × 10e6 PFU/mL), but resolving by 16 dpi (**Figure 3A**). In contrast to the rapid high titer achieved in pups, virus titers in adults inoculated with 400 PFU remained below the level of detection at 5 days (**Figure 3A**). To compare titers in a group that experienced significant lethality, we inoculated P8 pups i.n. with 10,000 PFU, which was the approximate LD50 at this postnatal age. Consistent with the low dose, inoculation at a high dose resulted in a rapid increase in virus titers peaking at 8 dpi (mean 2.6 × 10e7 PFU/mL) (**Figure 3B**).

Interestingly, virus titers did not differ extensively at 3, 5, or 16 dpi in P8 pups inoculated with a high- versus low-dose virus (**Figure 3C**). Notably, though, at the 8-day peak, lung titers in pups inoculated with 10,000 PFU virus were at a level nearly 10-fold higher than that of pups with low-dose inoculation. This high titer occurred at a time frame correlating with a fraction of the animals experiencing lethal disease (see **Figure 1A**). Together with the lethality results, these data demonstrate that neonatal MHV68 infection results in rapid virus replication in the lung, with very high titers correlating with lethal disease in a fraction of animals, but resolution of lytic replication in survivors.

### MHV68 infection in neonatal mice induces splenomegaly

**Figure 4:**
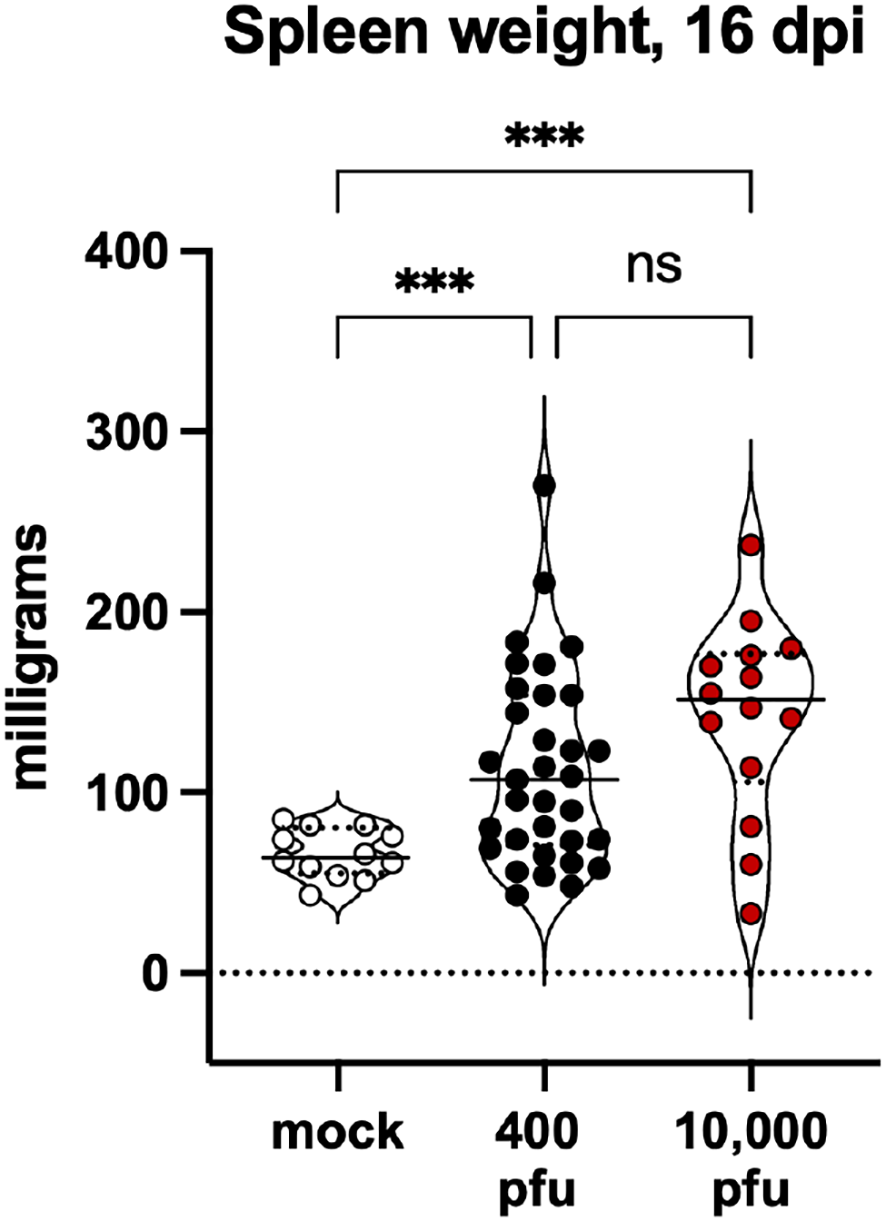
MHV68 infection during early life induces splenomegaly. P8 pups were mock-inoculated or inoculated with 400 PFU or 10,000 PFU MHV68. Spleens were harvested and weighed at 16 dpi. Each symbol represents an individual mouse (mock, n = 12; 400 PFU, n = 33; 10,000 PFU, n = 14). Significance was determined using Brown–Forsythe and Welch’s ANOVA with Dunnett’s T3 multiple comparisons. *p < 0.05; **p < 0.01; ***p < 0.001; ns, not significant. MHV68, murine gammaherpesvirus 68.

Adult C57BL/6J mice infected with MHV68 typically exhibit significant splenomegaly at 16 dpi as a result of robust expansion of Vβ4-restricted CD8 T cells (Tripp et al., 1997; Flano et al., 2004). To examine splenomegaly in the current cohort, we quantified spleen weights in P8 pups inoculated with 400 versus 10,000 PFU of virus. In both scenarios, mice displayed on average significantly increased spleen size as compared to the mock-infected controls (**Figure 4**). Although not statistically significant, high-dose inoculation resulted in a higher median weight as compared to low-dose inoculation (10,000 PFU, 151 mg; 400 PFU, 107 mg), with a significant fraction of the low-dose cohort exhibiting spleen sizes equivalent to those of mock-infected animals.

### Neonatal infection results in an increased frequency of latently infected cells during long-term infection as compared to adult infection

**Figure 5:**
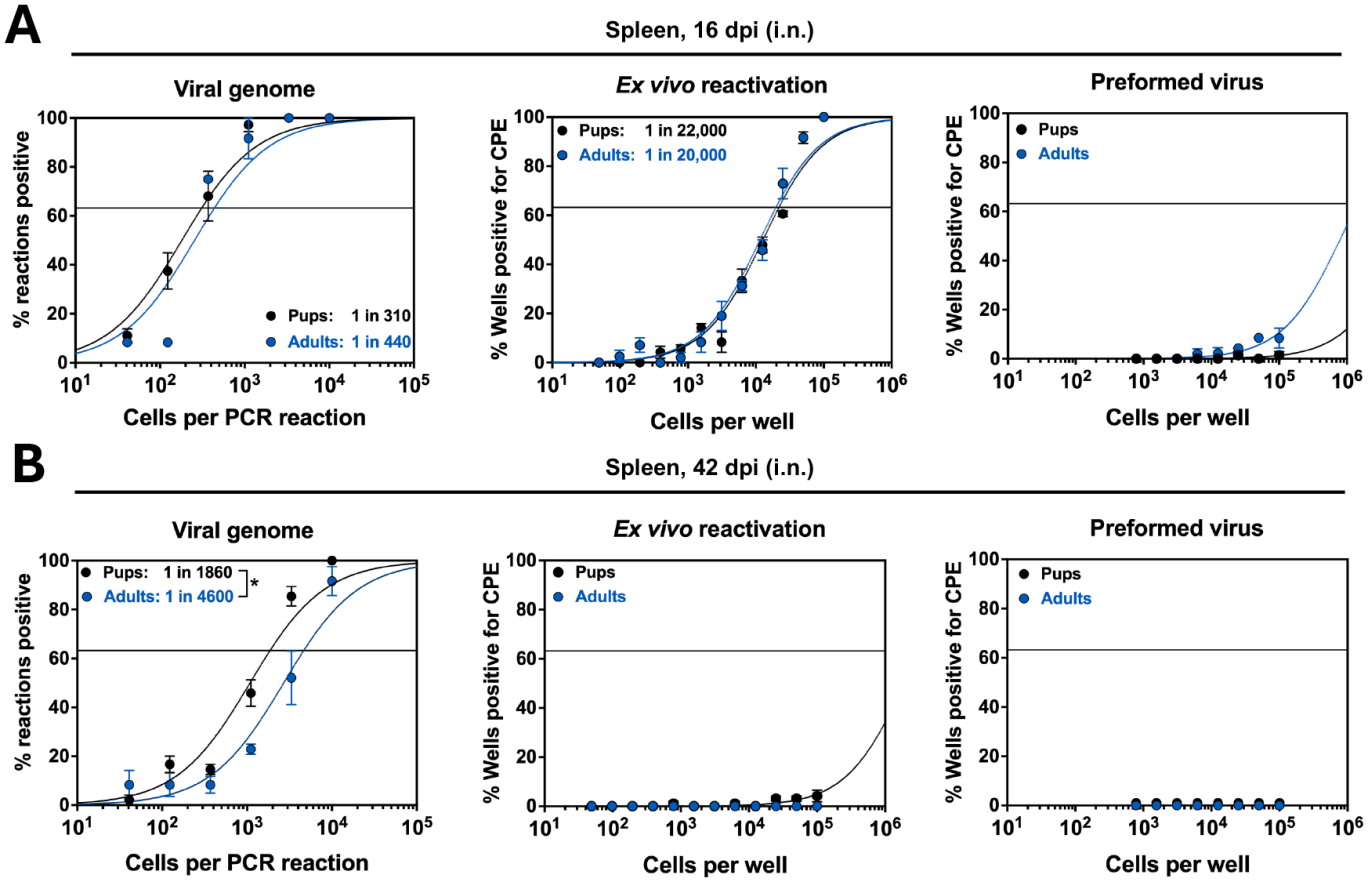
Early-life infection results in normal establishment of peak latency but increased long-term latency. P8 pups or 8- to 12-week-old adult mice were inoculated i.n. with 400 PFU MHV68. At **(A)** 16 dpi or **(B)** 42 dpi, splenocytes were harvested, pooled within experimental groups, and subjected to limiting dilution nested PCR to determine the frequency of cells carrying the MHV68 genome. Frequencies were determined using the Poisson distribution from non-linear regression, as indicated by the line at 63.2%. Parallel limiting dilution assays were performed to determine the frequencies of reactivation from latency and preformed infectious virus. Values represent mean ± SEM from independent experiments (16 days, n = 3; 42 days, n = 4). Significance was determined using a two-tailed, unpaired Student’s t-test. *p < 0.05; **p < 0.01; ns, not significant. MHV68, murine gammaherpesvirus 68; i.n., intranasal; dpi, days post-infection.

To determine whether early-life exposure to the virus results in altered latency establishment, parameters of latent infection were quantified at 16 dpi, a time that correlates with the resolution of the acute phase of lytic replication. To maximize survival, mice were i.n. inoculated at P8 with 400 PFU MHV68. Control adult mice were inoculated in parallel. Following harvest, single-cell suspensions of splenocytes were subjected to limiting dilution analyses of viral genome, reactivation from latency, and preformed infectious virus. At 16 dpi, the frequency of splenocytes carrying viral genome was similar (P8 pups, 1 in 310 cells; adults, 1 in 440 cells) between mice inoculated as P8 pups and mice inoculated as adults (**Figure 5A**). Parallel *ex vivo* reactivation and preformed virus assays demonstrated no significant difference between the P8 and adult groups. Furthermore, the normal level of reactivation coupled with low to undetectable levels of preformed virus indicated that virus genome-positive cells were latently infected. Together, these data indicate that neonatal infection results in the establishment of latency at a frequency nearly identical to that of adults.

A significant question in the field is whether early-life EBV and KSHV infection alters the long-term burden of latently infected cells (Guy de The, 1977; De-The et al., 1978; Rochford, 2016). In adult mice, the frequency of latently infected cells typically reaches peak levels around 16 days, then contracts over time, reaching significantly lower maintenance levels by 42 days and a stable long-term level by 90 days (Willer and Speck, 2003). Remarkably, this latency set point is not affected by virus dose at the time of inoculation (Tibbetts et al., 2003a). To characterize latency maintenance following neonatal infection, we first analyzed the frequency of splenocytes carrying the viral genome at 42 dpi. As expected, mice inoculated as adults displayed a lower frequency of latently infected cells as compared to 16 dpi, at a level of 1 in 4,600 splenocytes (**Figure 5B**). Consistent with a transition to long-term maintenance of latency, both groups displayed a low frequency of *ex vivo* reactivation (**Figure 5B**) and resolved the early splenomegaly observed at 16 dpi (not shown). Notably, though, mice inoculated in early life exhibited a 2.5-fold higher frequency of latently infected splenocytes (1 in 1,870).

**Figure 6:**
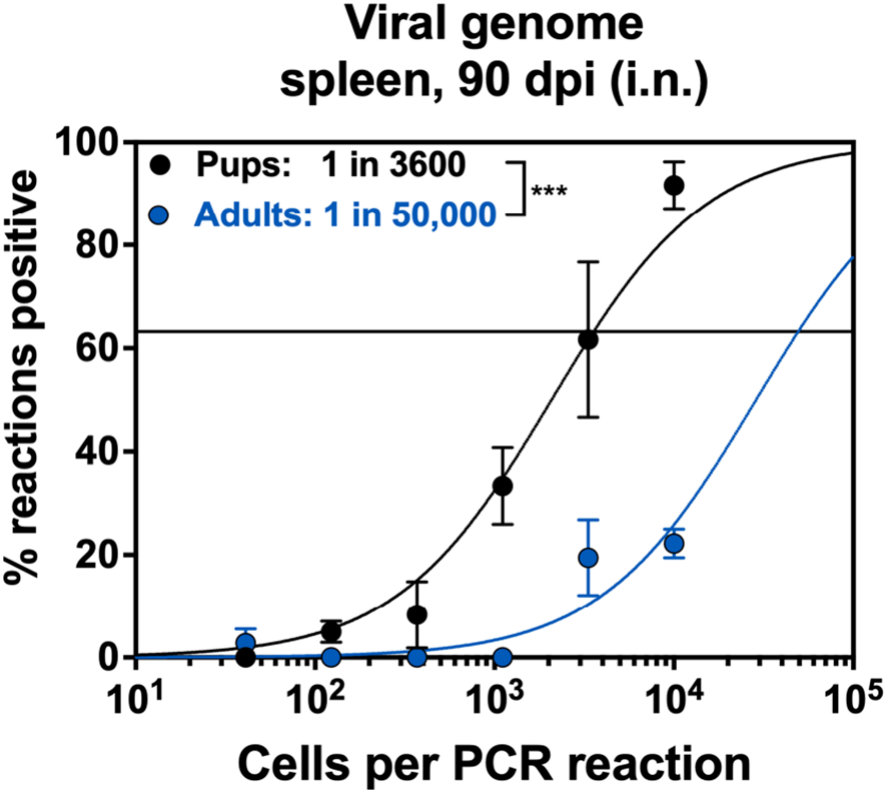
Early-life infection results in an expanded long-term latent reservoir. P5 pups or 8- to 12-week-old adult mice were inoculated i.n. with 200 PFU MHV68. At 90 dpi, splenocytes were harvested, pooled within experimental groups, and subjected to limiting dilution nested PCR to determine the frequency of cells carrying the MHV68 genome. Frequencies were determined using the Poisson distribution from non-linear regression, as indicated by the line at 63.2%. Values represent mean ± SEM from independent experiments (pups, n = 6; adults, n = 3). Significance was determined using a two-tailed, unpaired Student’s t-test. *p < 0.05; **p < 0.01; ***p < 0.001; ****p < 0.0001; ns, not significant. MHV68, murine gammaherpesvirus 68; i.n., intranasal; dpi, days post-infection.

Given this elevated frequency of latently infected cells in neonates versus adults at 42 days, we questioned whether exposure to the virus as a neonate may also result in elevated levels of latently infected cells during the stable maintenance phase of latency. To test this possibility, we infected adult and P5 neonatal mice in parallel and harvested spleens at 90 dpi. P5 pups were chosen for this long-term experiment in an effort to increase the period of time in which latency establishment would occur in the context of a developing immune response (due to the accelerated rate of development observed in mice as compared to humans). A lower dose of 200 PFU virus rather than 400 PFU was used in order to limit lethality. As expected for mice infected as adults, the frequency of latently infected cells dropped to stable long-term levels by 90 dpi, resulting in a frequency of approximately 1 in 50,000 splenocytes carrying the viral genome (**Figure 6**). In contrast, mice infected as neonates exhibited a 14-fold higher frequency of latently infected cells (1 in 3,600) as compared to adults. Together, these data demonstrate that early-life exposure to a gammaherpesvirus results in a substantially increased latency load during stable long-term infection.

## Discussion

EBV is frequently acquired during early childhood (2 to 5 years), and early childhood infection has been associated epidemiologically with increased risk of pediatric gammaherpesvirus-associated malignancies in settings where additional cofactors, such as malaria or HIV infection, are prevalent. However, the question of whether infection during the earliest stages of life, in which the immune system is still developing, may influence latency maintenance and eventual pathogenic outcomes such as malignancy and autoimmune disease remains unknown. Human studies are limited in their ability to define how early-life exposure to the virus alters long-term infection and disease, particularly because viral burden is often measured at disease diagnosis, rather than longitudinally from the day of infection across long-term latency. The MHV68 system provides a robust experimental model in which age at inoculation, acute replication, latency establishment, and long-term latency maintenance can be directly measured. The increased long-term frequency of latently infected cells observed here provides, to our knowledge, the first experimental evidence that early-life exposure to a gammaherpesvirus substantially alters the long-term latency reservoir.

In the work presented here, neonatal mice demonstrated age- and dose-dependent susceptibility to MHV68 disease, with animals inoculated at postnatal day 3 displaying the greatest susceptibility to lethal disease, but animals inoculated as late as P8 also displayed susceptibility to lethal disease. Lethal disease was accompanied by substantial weight loss prior to death. Surviving animals also experienced weight loss, but recovered prior to or during the lethality window. Regardless of inoculation dose, infection resulted in rapid acute lytic replication in the lung, with infectious virus reaching high titers by 3 days and peaking by 8 days. Although peak titers in neonates were significantly higher than those in adults, acute replication was largely resolved by 16 days, consistent with the window of acute replication in adult mice. Similar to that in adults, latency load in neonates peaked at 16 days and decreased over time, indicating that early-life infection does not simply increase the peak level of latency establishment. Strikingly, though, as late as 90 dpi during the stable phase of chronic infection, mice inoculated as neonates exhibited a nearly 14-fold increase in the number of latently infected cells as compared to adults. Together, these findings demonstrate that infection during the neonatal period of life directly impacts the long-term gammaherpesvirus latency reservoir.

**Figure 7:**
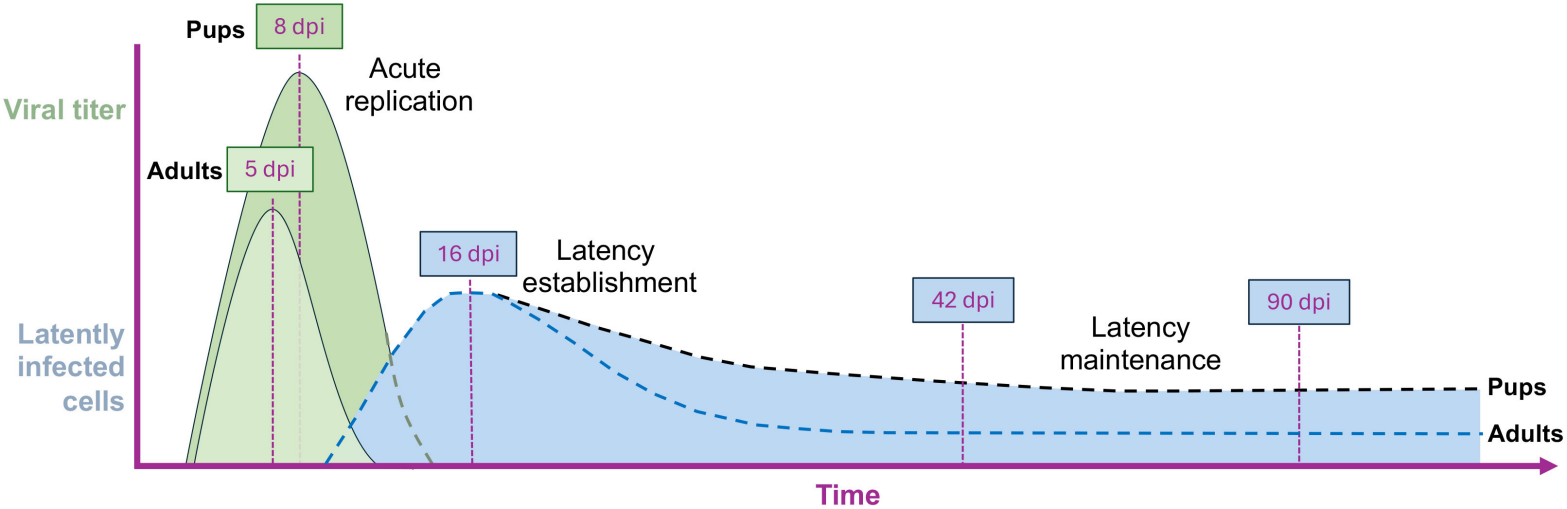
Early-life infection alters acute replication and long-term latency. Schematic comparing adult and neonatal MHV68 infection. Early-life infection results in increased lung lytic replication that peaks at 8 dpi and resolves by 16 dpi. Mice inoculated in early life and as adults exhibit similar peak latency loads during the establishment phase of latency, whereas mice inoculated during early life maintain an elevated frequency of latently infected cells during stable long-term latency. Y-axis represents the virus titer or frequency of latently infected cells for acute lytic replication or latency, respectively. X-axis represents time in days from initial inoculation to 90 days and throughout life. MHV68, murine gammaherpesvirus 68; dpi, days post-infection.

A central conclusion from these results is that early-life infection alters chronic latency without appreciably altering initial latency establishment. In adult mice, MHV68 latency in the spleen typically peaks near 16 dpi and subsequently contracts to a lower long-term set point. In the current study, mice infected as P8 pups displayed a frequency of viral genome-positive splenocytes at 16 dpi that was similar to that observed following adult infection. However, by 42 dpi, mice infected as neonates maintained a higher frequency of splenocytes carrying the viral genome than mice infected as adults. This difference was even more striking at 90 dpi, with mice infected as P5 pups maintaining a markedly higher frequency of viral genome-positive splenocytes than adult-infected controls. Although infected neonates also displayed significantly increased peak titers in the lung as compared to adults, this amplified replication phase is unlikely to be the direct cause of altered long-term latency, as the replication phase was resolved by 16 days and was not accompanied by a proportional increase in splenic latency at 16 days. Thus, the relationship between acute lytic burden and long-term latency is not explained solely by increased early seeding of the latency reservoirs. Thus, these data support a model in which neonatal infection alters the contraction and/or stable maintenance of the latent reservoir rather than increasing the initial establishment of splenic latency (**Figure 7**).

The current data do not distinguish whether this long-term effect is a function of altered viral latency processes, altered immune processes, or both. However, three scenarios would seem most likely to explain the reduced contraction of the latency reservoir following early-life infection: i) latency may be established in a population of cells that is accessible during early life but is not present in adults; ii) latency establishment occurs in expected cell types, but infection during this period alters the immune surveillance mechanisms that normally result in the contraction of the latency compartment; and iii) latency establishment during early life results in an increased proportion of cells in which latency is in a form with highly restricted viral gene expression, thereby preventing immune recognition.

Whether the cellular reservoir or anatomic compartment of latently infected cells is altered after early-life infection remains an important unresolved question. In adult hosts, both EBV and MHV68 are thought to exploit normal B-cell differentiation pathways to access the long-lived memory B-cell compartment. During neonatal infection, however, the virus encounters a developing immune system in which particular components, such as adaptive immunity, are still maturing. Thus, it is conceivable that, in the context of early-life infection, the virus accesses a latency establishment pathway and/or cell type that is in part distinct from that of adults, resulting in an expanded long-lived latency reservoir. For example, we have previously shown an infection of developing B cells in adults (Coleman et al., 2010; Coleman et al., 2014; Wang et al., 2024), and such cells are present in higher numbers during early life (Asito et al., 2011; Piriou et al., 2012). It is likewise conceivable that infection during early life allows the seeding of latency reservoirs in alternative tissues. Thus, future studies will be required to quantify and define virus-positive B-cell subsets and determine whether cellular reservoirs or anatomical compartments are durably altered following early-life exposure to the virus.

Whether the observed increase in the latency compartment may result from altered immune clearance also remains unknown. One immune factor that reflects an infection of adult mice is the induction of splenomegaly during latency establishment. This symptom of infection is in part a result of Vβ4-positive CD8 T-cell expansion, similar to the CD8 T-cell expansion that occurs during EBV mononucleosis (Usherwood et al., 1996; Tripp et al., 1997; Flano et al., 2004). In the work presented here, the infection of mice during the neonatal stage also resulted in splenomegaly, although to a lower extent than in adults (Usherwood et al., 1996), a finding that may reflect a lack of expansion of Vβ4-positive CD8 T cells, as was previously noted in neonates (Ptaschinski and Rochford, 2008). Nevertheless, the splenomegaly observed here suggests that mice infected as neonates do mount some form of immune response to infection. Additional studies will be required to determine whether this immune process is unique to early-life infection.

In light of our findings and the connection between EBV and autoimmune diseases such as multiple sclerosis (MS), the potential impact of early-life infection on immune tolerance and autoimmunity also deserves further examination. Neonatal infection resulting in immunotolerance has been postulated for other chronic infections, including hepatitis B virus (Hong and Bertoletti, 2017); however, this concept remains controversial, as other studies have shown that neonatal CD8 T cells in a lymphocytic choriomeningitis virus model display cell-intrinsic differences that increase their effector functions and resist exhaustion (Maymí et al., 2024). Nevertheless, the possibility of induced immune tolerance during early life gammaherpesvirus infections should also be considered.

The limitations of the current study should be noted, including the need for pooling of samples for latency assays and, therefore, a lack of individual mouse data during chronic infections; immune cell subsets have not yet been directly measured, and no causality for the expanded reservoir has as yet been determined.

Taken together, these studies establish neonatal MHV68 infection as a tractable model for defining how early-life gammaherpesvirus infection shapes chronic latency. The work here demonstrated that, as compared to adult infection, exposure to the virus during early life results in an increased susceptibility to severe acute disease and an expanded long-term latency reservoir. This model provides a foundation for future studies to define virus and host determinants that control latent reservoir size during early life and to test how the timing of primary infection during early life developmental stages contributes to gammaherpesvirus-associated diseases.

## Data Availability

The original contributions presented in the study are included in the article/supplementary material. Further inquiries can be directed to the corresponding author.

## References

[B63] Asito A. S. Piriou E. Jura W. G. Z. O. Ouma C. Odada P. S. Ogola S. . (2011). Suppression of circulating IgD+CD27+ memory B cells in infants living in a malaria-endemic region of Kenya. Malar. J. 10. doi: 10.1186/1475-2875-10-362 22166136 PMC3315680

[B26] Barton E. Mandal P. Speck S. H. (2011). Pathogenesis and host control of gammaherpesviruses: Lessons from the mouse. Annu. Rev. Immunol. 29, 351–397. doi: 10.1146/annurev-immunol-072710-081639 21219186

[B34] Basha S. Surendran N. Pichichero M. (2014). Immune responses in neonates. Expert Rev. Clin. Immunol. 10, 1171–1184. doi: 10.1586/1744666X.2014.942288 25088080 PMC4407563

[B10] Bhutani M. Polizzotto M. N. Uldrick T. S. Yarchoan R. (2015). Kaposi sarcoma-associated herpesvirus-associated Malignancies: Epidemiology, pathogenesis, and advances in treatment. Semin. Oncol. 42, 223–246. doi: 10.1053/j.seminoncol.2014.12.027 25843728 PMC6309362

[B7] Bjornevik K. Münz C. Cohen J. I. Ascherio A. (2023). Epstein–barr virus as a leading cause of multiple sclerosis: Mechanisms and implications. Nat. Rev. Neurol. 19, 160–171. doi: 10.1038/s41582-023-00775-5 36759741

[B40] Bonville C. A. Ptaschinski C. Percopo C. M. Rosenberg H. F. Domachowske J. B. (2010). Inflammatory responses to acute pneumovirus infection in neonatal mice. Virol. J. 7. doi: 10.1186/1743-422X-7-320 21078159 PMC2993675

[B13] Brady G. MacArthur G. J. Farrell P. J. (2008). Epstein-barr virus and Burkitt lymphoma. Postgrad. Med. J. 84, 372–377. doi: 10.1136/jcp.2007.047977 18716017

[B14] Caro-Vegas C. Peng A. Juarez A. Silverstein A. Kamiyango W. Villiera J. . (2023). Pediatric HIV+ kaposi sarcoma exhibits clinical, virological, and molecular features different from the adult disease. JCI Insight 8. doi: 10.1172/jci.insight.167854 37991023 PMC10721314

[B15] Chabay P. A. Preciado M. V. (2013). EBV primary infection in childhood and its relation to B-cell lymphoma development: A mini-review from a developing region. Int. J. Cancer 133, 1286–1292. doi: 10.1002/ijc.27858 23001576

[B61] Coleman C. B. McGraw J. E. Feldman E. R. Roth A. N. Keyes L. R. Grau K. R. . (2014). A gammaherpesvirus Bcl-2 ortholog blocks B cell receptor-mediated apoptosis and promotes the survival of developing B cells *in vivo*. PloS Pathog. 10, 13045–13052. doi: 10.1371/journal.ppat.1003916 24516386 PMC3916410

[B53] Coleman C. B. Nealy M. S. Tibbetts S. A. (2010). Immature and transitional B cells are latency reservoirs for a gammaherpesvirus. J. Virol. 84, 13045–13052. doi: 10.1128/jvi.01455-10 20926565 PMC3004345

[B47] Collins C. M. Boss J. M. Speck S. H. (2009). Identification of infected B-cell populations by using a recombinant murine gammaherpesvirus 68 expressing a fluorescent protein. J. Virol. 83, 6484–6493. doi: 10.1128/jvi.00297-09 19386718 PMC2698576

[B46] Collins C. M. Speck S. H. (2012). Tracking murine gammaherpesvirus 68 infection of germinal center B cells *in vivo*. PloS One 7. doi: 10.1371/journal.pone.0033230 22427999 PMC3302828

[B57] De-The G. Geser A. Day N. E. Tukeit P. M. Williams E. H. Beri D. P. . (1978). Epidemiological evidence for causal relationship between Epstein-Barr virus and Burkitt’s lymphoma from Ugandan prospective study. Nature 274, 756–761. doi: 10.1038/274756a0 210392

[B3] El-Mallawany N. K. McAtee C. L. Campbell L. R. Kazembe P. N. (2018). Pediatric kaposi sarcoma in context of the HIV epidemic in sub-saharan Africa: Current perspectives. Pediatr. Health Med. Ther. 9, 35–46. doi: 10.2147/phmt.s142816 29722363 PMC5919159

[B56] Flano E. Hardy C. L. Kim I.-J. Frankling C. Coppola M. A. Nguyen P. . (2004). T cell reactivity during infectious mononucleosis and persistent gammaherpesvirus infection in mice. J. Exp. Med. 196, 1363–1372. doi: 10.1084/jem.20020890 14978113

[B17] Guy de The (1977). Is burkitt’s lymphoma related to perinatal infection by Epstein-Barr virus? Lancet 309, 335–338. doi: 10.1016/s0140-6736(77)91137-0 64861

[B12] Hämmerl L. Colombet M. Rochford R. Ogwang D. M. Parkin D. M. (2019). The burden of Burkitt lymphoma in Africa. Infect. Agent Cancer 14. doi: 10.1186/s13027-019-0236-7 31388351 PMC6670145

[B44] Häusler M. Sellhaus B. Scheithauer S. Gaida B. Kuropka S. Siepmann K. . (2007). Myocarditis in newborn wild-type BALB/c mice infected with the murine gamma herpesvirus MHV-68. Cardiovasc. Res. 76, 323–330. doi: 10.1016/j.cardiores.2007.06.025 17658501

[B65] Hong M. Bertoletti A. (2017). Tolerance and immunity to pathogens in early life: Insights from HBV infection. Semin. Immunopathol. 39, 643–652. doi: 10.1007/s00281-017-0641-1 28685270 PMC5711997

[B36] IJspeert H. van Schouwenburg P. A. Zessen D. Pico-Knijnenburg I. Driessen G. J. Stubbs A. P. . (2016). Evaluation of the antigen-experienced B-cell receptor repertoire in healthy children and adults. Front. Immunol. 7. doi: 10.3389/fimmu.2016.00410 27799928 PMC5066086

[B2] Jackson C. C. Dickson M. A. Sadjadi M. Gessain A. Abel L. Jouanguy E. . (2016). Kaposi sarcoma of childhood: Inborn or acquired immunodeficiency to oncogenic HHV-8. Pediatr. Blood Cancer 63, 392–397. doi: 10.1002/pbc.25779 26469702 PMC4984265

[B19] Jayasooriya S. de Silva T. I. Njie-jobe J. Sanyang C. Leese A. M. Bell A. I. . (2015). Early virological and immunological events in asymptomatic Epstein-Barr virus infection in African children. PloS Pathog. 11, 1–18. doi: 10.1371/journal.ppat.1004746 25816224 PMC4376400

[B21] Kasolo F. C. Mpabalwani E. Gompels U. A. (1997). Infection with AIDS-related herpesviruses in human immunodeficiency virus-negative infants and endemic childhood Kaposi's sarcoma in Africa. J. Gen. Virol. 78, 847–856. doi: 10.1099/0022-1317-78-4-847 9129658

[B9] Leung A. K. C. Lam J. M. Barankin B. (2023). Infectious mononucleosis: An updated review. Curr. Pediatr. Rev. 20, 305–322. doi: 10.2174/1573396320666230801091558 37526456

[B5] Mamimandjiami A. I. Engone-Ondo J. D. Moussavou-Boundzanga P. Mouinga-Ondeme A. Mfouo-Tynga I. S. (2025). Kaposi’s sarcoma: A non-communicable outcome mainly prompted by communicable diseases in sub-saharan Africa. Int. J. Mol. Sci. 26. doi: 10.3390/ijms262010198 41155488 PMC12563117

[B66] Maymí V. I. Zhu H. Jager M. Johnson S. Getchell R. Casey J. W. . (2024). Neonatal CD8+ T cells resist exhaustion during chronic infection. J. Immunol. 212, 834–843. doi: 10.4049/jimmunol.2300396 38231127 PMC11298781

[B38] McCord J. L. Chatterjee D. Han J. Y. S. Scoles D. Smeyne R. J. Philp N. J. . (2026). Persistent microglial activation following neonatal CMV infection mediates neurodegeneration. Sci. Adv. 12 (11), eadz1686. doi: 10.1126/sciadv.adz1686 41811959 PMC12978234

[B24] Minhas V. Brayfield B. P. Crabtree K. L. Kankasa C. Mitchell C. D. Wood C. (2010). Primary gamma-herpesviral infection in Zambian children. BMC Infect. Dis. 10. doi: 10.1186/1471-2334-10-115 20462453 PMC2881090

[B1] Molyneux E. M. Rochford R. Griffi B. Newton R. Jackson G. Menon G. . (2012). Seminar burkitt’s lymphoma. Lancet 379, 1234–1278. doi: 10.1016/S0140-6736(11)61177-X 22333947

[B16] Moormann A. M. Chelimo K. Sumba O. P. Lutzke M. L. Ploutz-Snyder R. Newton D. . (2005). Exposure to holoendemic malaria results in elevated Epstein-Barr virus loads in children. J. Infect. Dis. 191, 1233–1238. doi: 10.1086/428910 15776368

[B25] Moormann A. M. Heller K. N. Chelimo K. Embury P. Ploutz-Snyder R. Otieno J. A. . (2009). Children with endemic Burkitt lymphoma are deficient in EBNAl-specific IFN-γ T cell responses. Int. J. Cancer 124, 1721–1726. doi: 10.1002/ijc.24014 19089927 PMC2708320

[B6] Motlhale M. Sitas F. Bradshaw D. Chen W. C. Singini M. G. de Villiers C. B. . (2022). Epidemiology of kaposi’s sarcoma in sub-saharan Africa. Cancer Epidemiol. 78. doi: 10.1016/j.canep.2022.102167 35504064

[B48] Nealy M. S. Coleman C. B. Li H. Tibbetts S. A. (2010). Use of a virus-encoded enzymatic marker reveals that a stable fraction of memory B cells expresses latency-associated nuclear antigen throughout chronic gammaherpesvirus infection. J. Virol. 84, 7523–7534. doi: 10.1128/jvi.02572-09 20484501 PMC2897616

[B37] Nielsen S. C. A. Roskin K. M. Jackson K. J. L. Joshi S. A. Nejad P. Lee J. Y. . (2019). Shaping of infant B cell receptor repertoires by environmental factors and infectious disease. Sci. Transl. Med. 11. doi: 10.1126/scitranslmed.aat2004 30814336 PMC6733608

[B39] Peiper A. M. Helm E. W. Nguyen Q. Phillips M. Williams C. G. Shah D. . (2023). Infection of neonatal mice with the murine norovirus strain WU23 is a robust model to study norovirus pathogenesis. Lab. Anim. (NY) 52, 119–129. doi: 10.1038/s41684-023-01166-5 37142696 PMC10234811

[B18] Piriou E. Asito A. S. Sumba P. O. Fiore N. Middeldorp J. M. Moormann A. M. . (2012). Early age at time of primary epstein-barr virus infection results in poorly controlled viral infection in infants from western Kenya: Clues to the etiology of endemic Burkitt lymphoma. J. Infect. Dis. 205, 906–913. doi: 10.1093/infdis/jir872 22301635 PMC3282570

[B43] Ptaschinski C. Rochford R. (2008). Infection of neonates with murine gammaherpesvirus 68 results in enhanced viral persistence in lungs and absence of infectious mononucleosis syndrome. J. Gen. Virol. 89, 1114–1121. doi: 10.1099/vir.0.83470-0 18420788

[B58] Rochford R. (2016). Epstein-Barr virus infection of infants: Implications of early age of infection on viral control and risk for Burkitt lymphoma. Bol. Med. Hosp. Infant Mex. 73, 41–46. doi: 10.1016/j.bmhimx.2015.12.001 29421232

[B23] Sabourin K. R. Daud I. Ogolla S. Labo N. Miley W. Lamb M. . (2021). Malaria is associated with Kaposi sarcoma-associated herpesvirus seroconversion in a cohort of western Kenyan children. J. Infect. Dis. 224, 303–311. doi: 10.1093/infdis/jiaa740 33249494 PMC8280487

[B22] Sabourin K. R. Ogolla S. Reyes G. S. Daud I. Jackson C. L. Labo N. . (2023). Effects of maternal HIV infection on early Kaposi sarcoma-associated herpesvirus seroconversion in a Ken an mother-infant cohort. J. Infect. Dis. 228, 1357–1366. doi: 10.1093/infdis/jiad310 37536370 PMC10640772

[B35] Semmes E. C. Chen J. L. Goswami R. Burt T. D. Permar S. R. Fouda G. G. (2021). Understanding early-life adaptive immunity to guide interventions for pediatric health. Front. Immunol. 11. doi: 10.3389/fimmu.2020.595297 33552052 PMC7858666

[B8] Shannon-Lowe C. Rickinson A. (2019). The global landscape of EBV-associated tumors. Front. Oncol. 9. doi: 10.3389/fonc.2019.00713 31448229 PMC6691157

[B30] Simas J. P. Efstathiou S. (1998). Murine gammaherpesvirus 68: A model for the study of gammaherpesvirus pathogenesis. Trends Microbiol. 6, 276–282. doi: 10.1016/s0966-842x(98)01306-7 9717216

[B20] Slyker J. A. Casper C. Tapia K. Richardson B. Bunts L. Huang M. L. . (2013). Clinical and virologic manifestations of primary Epstein-Barr virus (EBV) infection in Kenyan infants born to HIV-infected women. J. Infect. Dis. 207, 1798–1806. doi: 10.1093/infdis/jit093 23493724 PMC3654744

[B4444] Speck S. H. Virgin IV H. W. (1999). Host and viral genetics of chronic infection: A mouse model of gamma-herpesvirus pathogenesis. Curr. Opin. Immunol. 2, 403–409. doi: 10.1016/S1369-5274(99)80071-X 10458986

[B11] Stefan D. (2015). Patterns of distribution of childhood cancer in Africa. J. Trop. Pediatr. 61, 165–173. doi: 10.1093/tropej/fmv005 25724211

[B45] Štiglincová V. Chalupková A. Hrabovská Z. Čipková J. Wágnerová M. Mistríková J. (2011). Vertical transmission of murine gammaherpesvirus 68 in mice. Acta Virol. 55, 55–59. doi: 10.4149/av_2011_01_55 21434705

[B32] Sunil-Chandra N. P. Arno J. Fazakerley J. Nash A. A. (1994). Lymphoproliferative disease in mice infected with murine gammaherpesvirus 68. Am. J. Pathol. 145, 818–826. 7943173 PMC1887324

[B54] Sunil-Chandra N. P. Efstathiou S. Arno J. Nash A. A. (1992). Virological and pathological features of mice infected with murine gammaherpesvirus 68. J. Gen. Virol. 73, 2347–2356. doi: 10.1099/0022-1317-73-9-2347 1328491

[B31] Tarakanova V. L. Suarez F. Tibbetts S. A. Jacoby M. A. Weck K. E. Hess J. L. . (2005). Murine gammaherpesvirus 68 infection is associated with lymphoproliferative disease and lymphoma in BALB β2 microglobulin-deficient mice. J. Virol. 79, 14668–14679. doi: 10.1128/jvi.79.23.14668-14679.2005 16282467 PMC1287585

[B60] Tibbetts S. A. Loh J. van Berkel V. McClellan J. S. Jacoby M. A. Kapadia S. B. . (2003a). Establishment and maintenance of gammaherpesvirus latency are independent of infective dose and route of infection. J. Virol. 77, 7696–7701. doi: 10.1128/jvi.77.13.7696-7701.2003 12805472 PMC164792

[B50] Tibbetts S. A. McClellan J. S. Gangappa S. Speck S. H. Virgin H. W. (2003b). Effective vaccination against long-term gammaherpesvirus latency. J. Virol. 77, 2522–2529. doi: 10.1128/jvi.77.4.2522-2529.2003 12551990 PMC141097

[B55] Tripp R. A. Hamilton-Easton A. M. Cardin R. D. Nguyen P. Behm F. G. Woodland D. L. . (1997). Pathogenesis of an infectious mononucleosis-like disease induced by a murine-herpesvirus: Role for a viral superantigen? J. Exp. Med. 185, 1641–1650. doi: 10.1084/jem.185.9.1641 9151901 PMC2196306

[B64] Usherwood E. J. Ross A. J. Allen D. J. N A. A. (1996). Murine gammaherpesvirus-induced splenomegaly: a critical role for CD4 T cells. J. Gen. Virol. 77, 627–630. doi: 10.1099/0022-1317-77-4-627 8627250

[B500] Usherwood E. J. Ross A. J. Allen D. J. N A. A. (1996). Murine gammaherpesvirus-induced splenomegaly: a critical role for CD4 T cells. J. Gen. Virol. 77, 627–630. doi: 10.1099/0022-1317-77-4-627. Usherwood EJ, Ross AJ, Allen DJ, N AA. Murine gammaherpesvirus-induced splenomegaly: a critical role for CD4 T cells. (1996). 627–630 p. 8627250

[B51] Van Dyk L. F. Virgin I. H. Speck S. H . (2000). The murine gammaherpesvirus 68 v-cyclin is a critical regulator of reactivation from latency. J. Virol. 74, 7451–7461. doi: 10.1128/jvi.74.16.7451-7461.2000 PMC112265 10906198

[B5555] Virgin IV H. W. Speck S. H. (1999). Unraveling immunity to γ-herpesviruses: A new model for understanding the role of immunity in chronic virus infection. Curr. Opin. Immunol. 11, 371–379. doi: 10.1016/S0952-7915(99)80063-6 10448140

[B62] Wang Y. Feswick A. Apostolou V. Tibbetts S. A. (2024). The unappreciated role of developing B cells in chronic gammaherpesvirus infections. PloS Pathog. 20. doi: 10.1371/journal.ppat.1012445 39298520 PMC11412639

[B49] Wang Y. Manzi M. Feswick A. Renshaw L. Oliver P. M. Tibbetts S. A. . (2023). B cell expression of E3 ubiquitin ligase Cul4b promotes chronic gammaherpesvirus infection *in vivo*. J. Virol. 97. doi: 10.1128/jvi.01008-23 37962378 PMC10734415

[B27] Wang Y. Tibbetts S. A. Krug L. T. (2021). Annual review of virology conquering the host: Determinants of pathogenesis learned from murine gammaherpesvirus 68 Annual Rev. 8, 349–371. doi: 10.1146/annurev-virology-011921 PMC9153731 34586873

[B52] Weck K. E. Kim S. S. Virgin I. H. Speck S. H. (1999). B cells regulate murine gammaherpesvirus 68 latency. J. Virol. 73, 4651–4661. doi: 10.1128/JVI.73.6.4651-4661.1999 PMC112506 10233924

[B59] Willer D. O. Speck S. H. (2003). Long-term latent murine gammaherpesvirus 68 infection is preferentially found within the surface immunoglobulin D-negative subset of splenic B cells *in vivo*. J. Virol. 77, 8310–8321. doi: 10.1128/jvi.77.15.8310-8321.2003 12857900 PMC165249

[B41] Yin Z. Wu Y. Zhu R. Xu L. Lin Y. Yang H. . (2021). Development of a neonatal mouse model for coxsackievirus B1 antiviral evaluation. Virologica Sinica 36, 1575–1584. doi: 10.1007/s12250 PMC8476979 34581960

[B42] You D. Siefker D. T. Shrestha B. Saravia J. Cormier S. A. (2015). Building a better neonatal mouse model to understand infant respiratory syncytial virus disease. Respir. Res. 16. doi: 10.1186/s12931-015-0244-0 26231396 PMC4531813

[B4] Young L. S. Yap L. F. Murray P. G. (2016). Epstein-barr virus: More than 50 years old and still providing surprises. Nat. Rev. Cancer 16, 789–802. doi: 10.1038/nrc.2016.92 27687982

[B33] Yu J. C. Khodadadi H. Malik A. Davidson B. Salles É.S. Bhatia J. . (2018). Innate immunity of neonates and infants. Front. Immunol. 9. doi: 10.3389/fimmu.2018.01759 30105028 PMC6077196

